# Identifying the top predictors of student well-being across cultures using machine learning and conventional statistics

**DOI:** 10.1038/s41598-024-55461-3

**Published:** 2024-04-10

**Authors:** Ronnel B. King, Yi Wang, Lingyi Fu, Shing On Leung

**Affiliations:** 1 Department of Curriculum and Instruction, Faculty of Education, The Chinese University of Hong Kong, Hong Kong, China; 2 Faculty of Education, University of Macau, Taipa, Macau SAR China; 3 Department of Health and Kinesiology, College of Health, University of Utah, Salt Lake City, UT USA

**Keywords:** Subjective well-being, Programme for International Student Assessment, Machine learning, Life satisfaction, Positive affect, Negative affect, Psychology, Environmental impact

## Abstract

Alongside academic learning, there is increasing recognition that educational systems must also cater to students’ well-being. This study examines the key factors that predict adolescent students’ subjective well-being, indexed by life satisfaction, positive affect, and negative affect. Data from 522,836 secondary school students from 71 countries/regions across eight different cultural contexts were analyzed. Underpinned by Bronfenbrenner’s bioecological theory, both machine learning (i.e., light gradient-boosting machine) and conventional statistics (i.e., hierarchical linear modeling) were used to examine the roles of person, process, and context factors. Among the multiple predictors examined, school belonging and sense of meaning emerged as the common predictors of the various well-being dimensions. Different well-being dimensions also had distinct predictors. Life satisfaction was best predicted by a sense of meaning, school belonging, parental support, fear of failure, and GDP per capita. Positive affect was most strongly predicted by resilience, sense of meaning, school belonging, parental support, and GDP per capita. Negative affect was most strongly predicted by fear of failure, gender, being bullied, school belonging, and sense of meaning. There was a remarkable level of cross-cultural similarity in terms of the top predictors of well-being across the globe. Theoretical and practical implications are discussed.

## Introduction

Student well-being is associated with adaptive outcomes^1^. High levels of well-being are correlated with better motivation, greater engagement, and higher achievement^2,3^. Hence, a wide range of studies have explored the antecedents of well-being. Despite the number of studies that explored the factors associated with student well-being, most of these studies are beset by two key limitations. First, past studies focused mainly on how a limited range of variables shape well-being. However, well-being is a complex construct and is likely determined by many different factors^4,5^. Research that simultaneously includes a wider range of variables that facilitate, or thwart well-being is needed. Second, much of the research on well-being only focused on what researchers have called WEIRD (Western, Educated, Industrialized, Rich, Democratic) societies^6^. Although there is now a growing body of work on well-being across different parts of the globe, existing knowledge is still heavily dependent on data generated from WEIRD societies.

This study aims to examine the roles of a wide range of factors in understanding student well-being. To address the first limitation, we conducted an integrative examination of the different factors that could predict students’ well-being using Bronfenbrenner’s bioecological theory. In total, we included 37 different predictor variables and examined which factors emerged as the most important predictors of well-being. To address the second limitation, we leveraged the latest Program for International Student Assessment (PISA) data, which included more than half a million students residing in 71 countries/regions from different cultural contexts^7^.

### Subjective well-being

Subjective well-being (SWB) refers to how individuals feel and think about their lives^8^. It can be divided into different components. The first component is cognitive well-being, which involves one’s assessment of overall life satisfaction. The second component is affective, which reflects the presence of pleasant affect (e.g., feelings of happiness) and the absence of unpleasant affect (e.g., depressed mood). Affective well-being focuses on the emotional experiences and feelings that individuals have in their daily lives. Hence, subjective well-being is typically assessed as a tripartite construct comprised of *life satisfaction* (cognitive judgment of the quality of one’s life), *positive affect* (experiences of positive emotions such as joy and pride), and *negative affect* (experiences of negative emotions such as anger and anxiety)^9^.

These three dimensions of subjective well-being are correlated but also show some degree of independence. For example, positive and negative affect are not exact opposites but are moderately negatively correlated with each other^9^. Because subjective well-being is not a unitary construct, these three dimensions need to be assessed independently of each other.

There are different determinants of subjective well-being^10,11^. Some studies have emphasized the role of personality traits. For example, having high levels of extraversion and conscientiousness and low neuroticism were associated with higher well-being^12^. Other studies have focused on genetic factors. Genes linked to depression, extraversion, and neuroticism seem to be driving how genetic predispositions influence well-being^13,14^. Contextual factors have also been found to be closely associated with well-being^15^. For example, citizens in poor countries have substantially lower well-being than their counterparts in rich countries.

Similarly, students’ subjective well-being could also be affected by multiple factors. Previous studies have revealed that personal, family, school, and country factors may shape student well-being^4,5,8,16^. Studies have found personal attributes (e.g., resilience^17^), family involvement (e.g., parental support^18^), school characteristics (e.g., teacher support^19^), and country factors (e.g., income inequality^20^) play an important role. For example, prior research on student well-being using the PISA dataset found that demographic factors such as socioeconomic status and gender were correlated with well-being^21^. Specifically, students from more advantaged families and boys experienced higher levels of well-being. Other contextual factors have also been found to be important. For example, students who perceived higher levels of teacher support had a greater sense of school belonging. Conversely, those who were more exposed to bullying had lower levels of well-being^17,22^.

Although past studies have contributed to providing more insights into the factors associated with students’ well-being, a key concern has been the lack of research that took a more holistic perspective and simultaneously examined how these different factors played a role in subjective well-being. Most of them examined the factors in isolation from each other. We address this limitation in the current study.

### Bronfenbrenner’s bioecological theory

To address the role of multiple factors in well-being, Bronfenbrenner’s bioecological theory was used. It is one of the most prominent and comprehensive frameworks that can be used to understand human functioning^23^. It has also been used in prior research to examine well-being^24^. The bioecological theory focuses on the role of four key factors that shape human development: proximal processes, person, context, and time^20,25–27^. We elucidate these factors below.

### Proximal processes

Proximal processes involve reciprocal interactions between individuals and their social partners (i.e., people, symbols, tasks, and objects). They are the primary mechanisms of interactions between humans and the environment. In this study, proximal processes pertain to how students engage with their learning materials and academic activities, as demonstrated by their usage of meta-cognitive strategies (i.e., summarizing and understanding, memorizing, and assessing credibility)^7,28^. Meta-cognitive strategies have been found to be positively associated with subjective well-being in previous studies^29,30^.

### Person factors

Person factors include innate characteristics such as demographic characteristics (e.g., gender), personality traits, motivation, and attitudes. Gender has been linked to well-being, with girls experiencing higher negative affect than boys^31,32^. Goals and aspirations are also key factors in understanding students’ well-being^33,34^. Individuals who made better progress toward their goals or those who are able to realize their aspirations have higher levels of well-being than others^34^.

Other psychological factors such as self-efficacy when facing adversity, fear of failure, competitiveness, as well as self-concept of task difficulty and competence might also be closely associated with well-being. Together, these concepts capture students’ beliefs in their capability to cope with adversity and other challenging situations^35–37^. Self-efficacy and self-concept have positive associations with well-being^36,38^, while fear of failure undermines well-being^39,40^.

### Context factors

Context pertains to the physical and social environment and can be further divided into microsystem, mesosystem, exosystem, and macrosystem levels. The microsystem is the immediate setting in which an individual lives and can include the family and school contexts. The mesosystem refers to the interconnections between different components of the microsystem (e.g., the connection between a child’s family and the school). The exosystem includes contexts that indirectly impact the individual’s development even if he/she is not directly participating in it. For example, a parent’s workplace can impact a child’s socio-emotional adjustment. The broadest is the macrosystem which includes the broader socioeconomic, cultural, and ideological patterns that shape an individual’s development.

In this study, we focus specifically on microsystem and macrosystem factors, as most of the contextual variables in the PISA dataset are located at these systems. Examples of microsystem factors in the PISA dataset include family-related factors such as parental support. Previous studies have found that parental support is a crucial factor for students’ well-being. Supportive parents cultivate their children’s sense of autonomy, are more supportive of their children’s schoolwork, and enjoy warm relationships with their children, all of which help facilitate well-being^18^. Parental support is also associated with higher levels of positive affect and lower levels of negative affect^41^.

Other microsystem factors include school climate and school resources. The school climate refers to the school atmosphere (i.e., school belonging, bullying, competitiveness, cooperation, and disciplinary climate), and the teaching and learning environment (i.e., teaching support, teacher-directed instruction, feedback, teachers’ stimulation, and adaptation of instruction). Among these factors, school belonging is related to more frequent positive feelings, fewer emotional problems, and greater subjective well-being^42,43^. Conversely, the experience of being bullied can undermine students’ subjective well-being^44^. Competitive school climates have been found to be associated with a higher frequency of mental health problems among students^39^, while climates characterized by cooperation are associated with higher levels of student well-being^43^.

Macrosystem factors include family socioeconomic status which includes elements such as family income^45^, parental level of education^46^, and parents’ occupation^47,48^. Previous studies have found that students from more disadvantaged families experience more stress and lower levels of well-being^49,50^.

Other macrosystem factors include country-level factors such as country affluence indexed in terms of Gross Domestic Product (GDP) per capita and national income inequality, typically indexed using the Gini coefficient. Previous studies have found that individuals in wealthier countries have higher levels of happiness and life satisfaction^51^. Income inequality, on the other hand, has been found to be associated with maladaptive outcomes, such as low school belonging, high test anxiety, and poor academic performance^52,53^. Studies have also found that subjective well-being is lower among individuals in unequal societies^54^.

### Time

Time incorporates multiple time scales of development and captures individuals’ trajectories. Time was not included in this study due to the cross-sectional nature of the PISA dataset. It is important to note, however, that PISA focuses on 15-year-old adolescent students, and the findings of this study are situated within this developmental stage.

Despite some cross-cultural differences, the expectations for adolescent students across the globe share certain similarities^55^. Adolescent students are expected to do well in school and prepare for either going into higher education or joining the workforce after secondary education. Adolescence is also a critical period for social and emotional development, and students are expected to develop healthy relationships and self-awareness, while navigating the biological and social changes associated with puberty. These societal expectations could shape adolescents’ well-being.

### Cultural similarities and differences

Well-being varies across cultures^56^. However, much of the current research on well-being has mostly relied on Western samples. Culture involves a rich complexity of “meanings, beliefs, practices, symbols, norms, and values prevalent among people in a society”^57^. Schwartz proposed the Cultural Values Theory to explore how different cultures vary in terms of their value orientations^57^. He proposed that different societies across the world can be categorized into eight distinct cultures based on how they prioritize cultural values.

The first dimension of cultural value contrasts *autonomy* (emphasis on creativity, curiosity, self-expression, pleasure, and enjoyment) with *embeddedness* (emphasis on social hierarchy, authority, and respect for tradition). The second dimension contrasts *hierarchy* (emphasis on social hierarchy, authority, and tradition) with *egalitarianism* (emphasis on equality, fairness, and justice), and the third dimension contrasts *mastery* (emphasis on achievement, success, and competence) with *harmony* (emphasis on social relationships, mutual respect, and consensus).

Based on how countries prioritize different cultural values, they can be classified into eight cultural groups: Africa and the Middle East, Confucian, East-Central Europe, East Europe, English Speaking, Latin America, Southeast Asia, and West Europe^58^. For example, Confucian Asia (e.g., China) is high in embeddedness, hierarchy, and mastery. Countries in Africa and the Middle East (e.g., Nigeria) score higher in embeddedness and have lower scores in mastery and autonomy.

### Explanation and prediction paradigms

In analyzing the data for this study, we use both the explanation and prediction paradigms. Explanation focuses on describing the causal relationships among variables by drawing on specific theoretical models. Conventional statistics is typically rooted in the explanation paradigm. It is usually grounded in a parsimonious theoretical model and can be used to explore the relationship between the independent and dependent variables^40,42^. Conventional statistics has the advantage of generating interpretable parameter estimates. For example, one can use conventional statistics (e.g., linear regression) to estimate the direction and magnitude strength of the association between a predictor (X) and an outcome variable (Y). The researcher can input data for the independent and dependent variables into the regression model and generate a parameter estimate that captures the direction and magnitude of the association between X and Y.

Machine learning, on the other hand, is rooted in the prediction paradigm. It does not generate parameter estimates and is a ‘black box’. Instead, machine learning focuses on identifying the most powerful predictors of the outcome variables. For example, a researcher using machine learning can input 100 predictors into the model and let the machine identify which among the variables best predict the outcome. By leveraging advanced algorithms, machine learning enables researchers to delve into large-scale datasets and uncover patterns in the data that would otherwise not have been possible with conventional statistics^59^.

Compared to conventional statistical methods, machine learning methods provide flexibility for modeling a large number of predictors and complex associations (i.e., nonlinearity and interaction) between predictors and outcomes^59^. Unlike conventional statistics, it can handle highly correlated predictors. In addition, machine learning involves splitting the data into a training set and a validation set. This maximizes the generalizability of findings to new data, optimizes predictive accuracy, and reduces problems of overfitting^60^. However, machine learning results are not readily interpretable, as they do not generate interpretable parameter estimates such as beta coefficients. Hence, in this study, we aimed to use both machine learning and conventional statistical analyses.

### The present study

In the current study, we aimed to (1) identify the most important predictors of students’ subjective well-being using machine learning approaches (*prediction*) and (2) explore how these predictors contributed to explaining variance in students’ subjective well-being using conventional statistics (*explanation*). Hence, we drew on both the prediction paradigm of machine learning and the explanation paradigm of conventional statistics and leveraged the strengths of both approaches.

**Figure 1:**
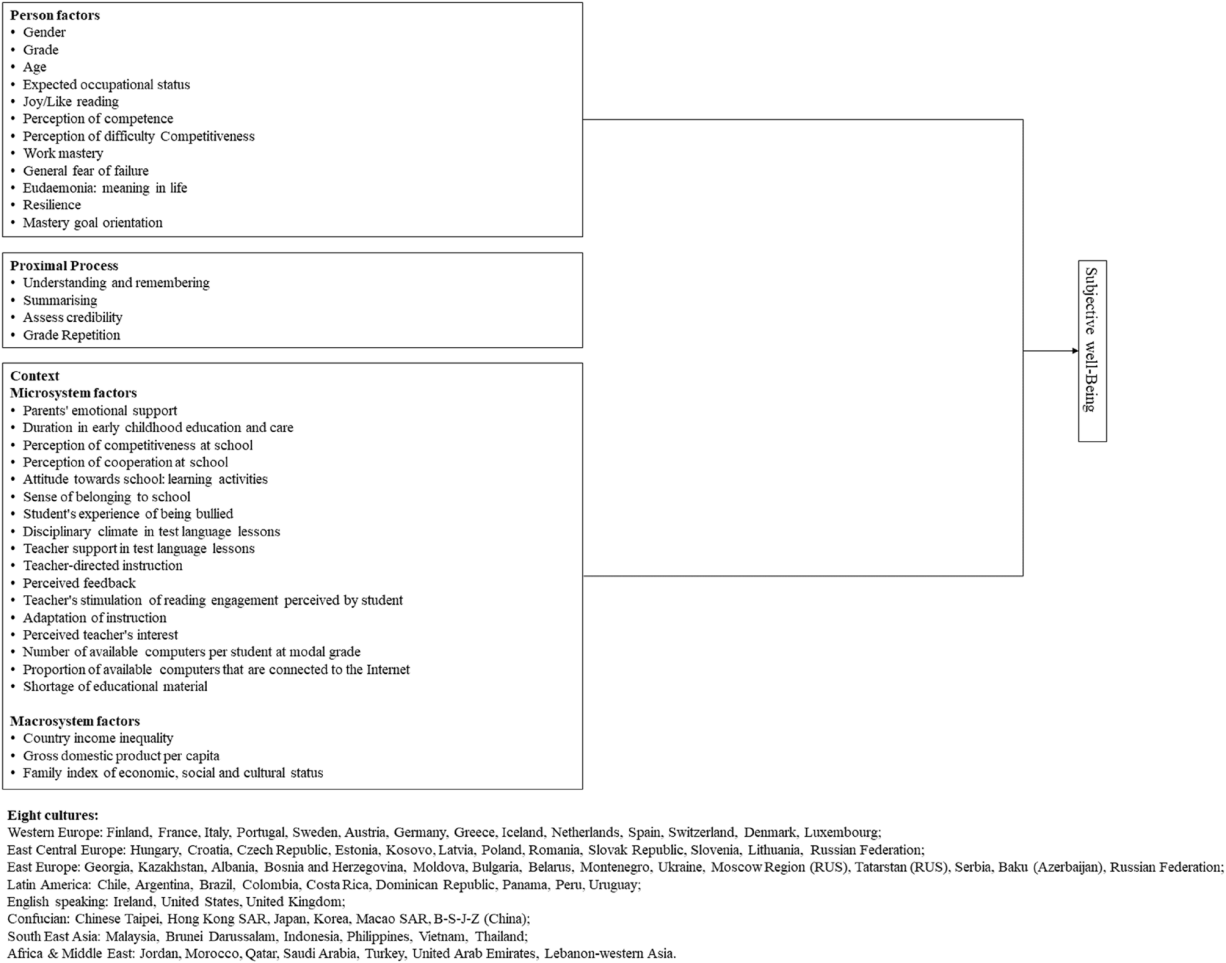
Conceptual model for the current study.

We also examined how the patterns of relationship between the predictors and subjective well-being outcomes were similar or different across cultural contexts (i.e., Africa and the Middle East, Confucian, East-Central Europe, East Europe, English Speaking, Latin America, Southeast Asia, and West Europe). The conceptual framework for the present study is shown in Fig. 1.

## Methods

### Data

This study drew on the Programme for International Student Assessment (PISA) 2018 data (Available at https://www.oecd.org/pisa/data/). The participants include 522,836 15-year-old students (*M* = 15.79, *SD* = 0.29) from 71 countries/regions. All countries/regions were divided into eight cultural groups based on Schwartz’s model. Table 1 shows the countries and sample size of each cultural group. Ethical approval was not required for this study as we used secondary analyses of existing data that is publicly available and de-identified.

**Table 1:** Countries/regions within each culture.

Culture	Sample size	Number of countries/regions	Ratio of females (%)	Countries/regions within each cultural group
Western Europe	119,910	15	49.2	Finland, France, Italy, Portugal, Sweden, Austria, Germany, Greece, Iceland, Netherlands, Spain, Switzerland, Denmark, Luxembourg, Malta
East Central Europe	64,388	11	49.5	Hungary, Croatia, Czech Republic, Estonia, Kosovo, Latvia, Poland, Romania, Slovak Republic, Slovenia, Lithuania
East Europe	95,922	14	51.6	Georgia, Kazakhstan, Albania, Bosnia and Herzegovina, Moldova, Bulgaria, Belarus, Montenegro, Ukraine, Moscow Region (RUS), Tatarstan (RUS), Serbia, Baku (Azerbaijan), Russian Federation
Latin America	68,323	9	50.9	Chile, Argentina, Brazil, Colombia, Costa Rica, Dominican Republic, Panama, Peru, Uruguay
English-speaking	24,233	3	50.1	Ireland, United States, United Kingdom
Confucian	41,872	6	49.0	Chinese Taipei, Hong Kong SAR, Japan, Korea, Macao SAR, B-S-J-Z (Mainland China)
Southeast Asia	46,280	6	52.1	Malaysia, Brunei Darussalam, Indonesia, Philippines, Vietnam, Thailand
Africa and Middle East	61,908	7	49.4	Jordan, Morocco, Qatar, Saudi Arabia, Turkey, United Arab Emirates, Lebanon-Western Asia

B–S–J–Z refers to Beijing–Shanghai–Jiangsu–Zhejiang which are all part of Mainland China. SAR refers to Special Autonomous Region.

### Subjective well-being

Subjective well-being was the key dependent variable. It was operationalized in terms of students’ life satisfaction, positive affect, and negative affect. Students were asked about their overall life satisfaction with one item (i.e., “Overall, how satisfied are you with your life as a whole these days”). This item was rated from 0 to 10, with higher numbers representing a higher level of life satisfaction.

Positive and negative affect were operationalized as how they generally feel in their lives, using five positive adjectives (e.g., joyful) and four negative adjectives (e.g., afraid), each of which was rated on a 4-point Likert scale (1 = *Never* to 4 = *Always*). The internal consistencies for positive affect (Cronbach's α = 0.79) and negative affect (Cronbach's α = 0.74) were acceptable.

### Predictors

Based on Bronfenbrenner’s bioecological theory^23^, 37 variables were selected from the PISA dataset as predictor variables (see Table 2 for detailed descriptions of all variables). These predictors were based on the PISA Assessment and Analytical Framework created by the OECD^7^ (see https://www.oecd.org/education/pisa-2018-assessment-and-analytical-framework-b25efab8-en.htm). PISA encompasses many items/variables related to students', parents', and schools' characteristics. Using these items/variables, OECD calculated derived variables based on item response theory (IRT) scaling. Given that the focus of PISA 2018 was on student well-being, many of the variables in the database were specifically selected by the OECD because of their theoretical linkages to well-being in the existing literature.

**Table 2:** Descriptive statistics, variable description, and bivariate correlations with subjective well-being.

Label	Description	Bioecological theory category	Mean	SD	*r* with life satisfaction	*r* with positive affect	*r* with negative affect
Outcome variables							
Life satisfaction	Cognitive well-being: overall life satisfaction	Individual	7.19	2.53	1		
Positive affect	Affective well-being: frequency of positive emotions	Individual	3.22	0.56	0.490**	1	
Negative affect	Affective well-being: frequency of negative emotions	Individual	2.36	0.60	− 0.326**	− 0.185**	1
Predictors							
Gender	Gender	Individual	1.50	0.50	0.074**	0.007**	− 0.208**
GRADE	Grade compared to modal grade in country	Individual	− 0.19	0.65	− 0.008**	0.005**	0.005**
AGE	Age	Individual	15.79	0.29	− 0.008**	− 0.009**	− 0.001
BSMJ	Student’s expected occupational status	Individual	65.26	17.95	0.024**	0.052**	0.040**
UNDREM	Meta-cognition: understanding and remembering	Proximal process	− 0.11	0.97	− 0.020**	− 0.010**	0.041**
METASUM	Meta-cognition: summarising	Proximal process	− 0.21	0.98	− 0.025**	− 0.015**	0.046**
METASPAM	Meta-cognition: assess credibility	Proximal process	− 0.22	0.94	− 0.051**	− 0.064**	0.060**
JOYREAD	Joy/like reading	Individual	0.17	0.99	0.015**	0.039**	0.093**
SCREADCOMP	Self-concept of reading: perception of competence	Individual	− 0.06	0.92	0.097**	0.149**	− 0.036**
SCREADDIFF	Self-concept of reading: perception of difficulty	Individual	0.09	0.94	− 0.070**	− 0.055**	0.125**
COMPETE	Competitiveness	Individual	0.05	0.97	0.088**	0.182**	− 0.044**
WORKMAST	Work mastery	Individual	0.08	0.99	0.179**	0.259**	− 0.034**
GFOFAIL	General fear of failure	Individual	− 0.05	0.94	− 0.208**	− 0.142**	0.328**
EUDMO	Eudaimonia: sense of meaning in life	Individual	0.14	0.94	0.399**	0.407**	− 0.194**
RESILIENCE	Resilience	Individual	0.02	0.98	0.284**	0.401**	− 0.170**
MASTGOAL	Mastery goal orientation	Individual	0.07	1.02	0.229**	0.295**	− 0.055**
REPEAT	Grade Repetition	Proximal process	0.54	1.91	− 0.062**	− 0.029**	0.058**
EMOSUPS	Parents' emotional support perceived by student	Context	− 0.08	0.95	0.259**	0.300**	− 0.091**
DURECEC	Duration in early childhood education and care	Context	2.66	1.16	0.005**	− 0.037**	0.001
ESCS	Index of economic, social and cultural status	Context	− 0.35	1.11	0.031**	0.018**	− 0.020**
PERCOMP	Perception of competitiveness at school	Context	0.04	0.92	0.053**	0.127**	0.019**
PERCOOP	Perception of cooperation at school	Context	− 0.01	0.94	0.210**	0.261**	− 0.079**
ATTLNACT	Attitude towards school: learning activities	Context	− 0.02	0.99	0.127**	0.149**	− 0.019**
BELONG	Sense of belonging to school	Context	− 0.08	0.94	0.273**	0.340**	− 0.218**
BEINGBULLIED	Student's experience of being bullied	Context	0.10	0.98	− 0.182**	− 0.169**	0.173**
DISCLIMA	Disciplinary climate in test language lessons	Context	0.15	1.08	0.149**	0.132**	− 0.094**
TEACHSUP	Teacher support in test language lessons	Context	0.18	0.97	0.158**	0.165**	− 0.060**
DIRINS	Teacher-directed instruction	Context	0.27	1.04	0.161**	0.159**	− 0.076**
PERFEED	Perceived feedback	Context	0.09	0.98	0.128**	0.156**	− 0.055**
STIMREAD	Teacher's stimulation of reading engagement perceived by student	Context	0.14	1.02	0.147**	0.178**	− 0.034**
ADAPTIVITY	Adaptation of instruction	Context	0.05	0.99	0.131**	0.152**	− 0.041**
TEACHINT	Perceived teacher's interest	Context	0.11	0.98	0.171**	0.203**	− 0.039**
RATCMP1	Number of available computers per student at modal grade	Context	0.68	0.85	− 0.026**	− 0.029**	0.021**
RATCMP2	Proportion of available computers that are connected to the Internet	Context	0.89	0.26	− 0.046**	− 0.038**	0.023**
EDUSHORT	Shortage of educational material	Context	0.13	1.08	0.030**	0.014**	− 0.012**
GINI	country income inequality	Context	35.17	6.75	− 0.030**	0.036**	0.076**
GDP per capita	Gross domestic product per capita	Context	25,439.36	22,379.30	− 0.084**	− 0.055**	0.045**

**p < 0.001, *r* pertains to the correlation coefficient between the predictor and the outcome variables. All the labels, except for GINI and GDP per capita, were derived from the PISA Assessment and Analytical Framework.

To make the result comparable across countries/regions, these variables were scaled using the OECD mean scores, calculated by PISA, with a standard deviation of − 1 to + 1 (see PISA 2018 technical report for further details: https://www.oecd.org/pisa/data/pisa2018technicalreport/PISA2018_Technical-Report-Chapter-16-Background-Questionnaires.pdf).

Two additional country factors (i.e., Gini and GDP per capita) were used from the World Bank website (https://www.worldbank.org/en/home). The Cronbach's alpha internal reliability values of these independent variables ranged from 0.64 to 0.91.

### Analysis

In the preliminary analysis, we excluded 9 countries that had high rates of missing data, ranging from 18.9% to 44.0%. The excluded countries were Norway, Belgium, North Macedonia, Mexico, Australia, New Zealand, Canada, Singapore, and Israel. Next, we clustered the remaining 71 countries/regions into Schwartz’s eight cultural groups^58^. Missing data were imputed using the *missForest* package^61^ in Python 3.8.8^62^.

The primary analyses consisted of two steps, with the first step relying on machine learning and the second step using conventional statistics. The Python syntax for both the machine learning and conventional statistical analyses can be found in the Supplementary Materials.

#### Step 1: machine learning

To address the first research objective of identifying the most important predictors of students’ subjective well-being, we used a machine learning algorithm to model the three elements of subjective well-being. The *scikit-learn* package was used to perform five tree-based ensemble machine learning algorithms to identify the top predictors of well-being. We used different algorithms including gradient boosted decision tree (GBDT), adaptive boosting (AdaBoost), ExtraTrees (ET), RandomForest (RF), and light gradient-boost machine (LightGBM). We compared the predictive accuracy of these five algorithms and selected the best among them. Mean Square Error (MSE) was used to determine the prediction accuracy of the model. Mean Absolute Error (MAE) was used to evaluate the differences between the prediction and true value. Lower MSE and MAE values indicate a higher rate of model accuracy. The coefficient of determination (*R*^2^) explains the amount of variance in well-being accounted for by the predictors.

Among the different machine learning algorithms, LightGBM performed better than others with the lowest MSE and MAE values and the highest *R*^2^ (see Table S1 in the supplementary file for more details). Therefore, we used the LightGBM algorithm as the primary analytic method in the first step. A tenfold cross-validation with 10 repeats was performed to streamline the models and select the top factors that have the strongest power for predicting well-being. For a better interpretation of the LightGBM model, we used the Shapley Additive exPlanations (SHAP) values that evaluate the contribution of each factor, not just the quality of the prediction itself^63^.

#### Step 2: conventional statistics

To address the second objective of exploring how much variance in well-being was explained by the different predictors, we used conventional statistics. More specifically, hierarchical linear modeling (HLM) was conducted due to the nested nature of the data as the students were nested within schools, which were nested within countries/regions^64^. Life satisfaction, positive affect, and negative affect were the outcome variables.

The top predictors identified by LightGBM were designated as the predictor variables. Hence, rather than using all 37 predictors, we only used those predictors that emerged as important in Step 1. We calculated the fixed and random effects of all top factors at level 1. Random effects of schools and countries/regions were at level 2 and level 3, respectively. The value of the intraclass correlation coefficient (ICC) was used to examine the percentage of the variance in subjective well-being explained by the school and/or country level. The equations for the HLM models can be found in the Supplementary Materials (see Eq. S1).

#### Supplementary analysis

Supplementary analyses were also conducted to determine whether the results were similar or different across cultures. We analyzed the results separately for each of the eight cultural contexts.

## Results

### Preliminary analyses

The descriptive statistics, variable description, and correlations with well-being for the overall sample can be seen in Table 2. The bivariate correlations among all variables are shown in Table S3 in the supplementary file.

### Step 1: machine learning

The LightGBM regression model with 37 predictors was used as it performed better than the other machine learning algorithms such as GBDT, AdaBoost, ET, and RF. The comparison among the different machine learning algorithms can be found in Table S1.

**Figure 2:**
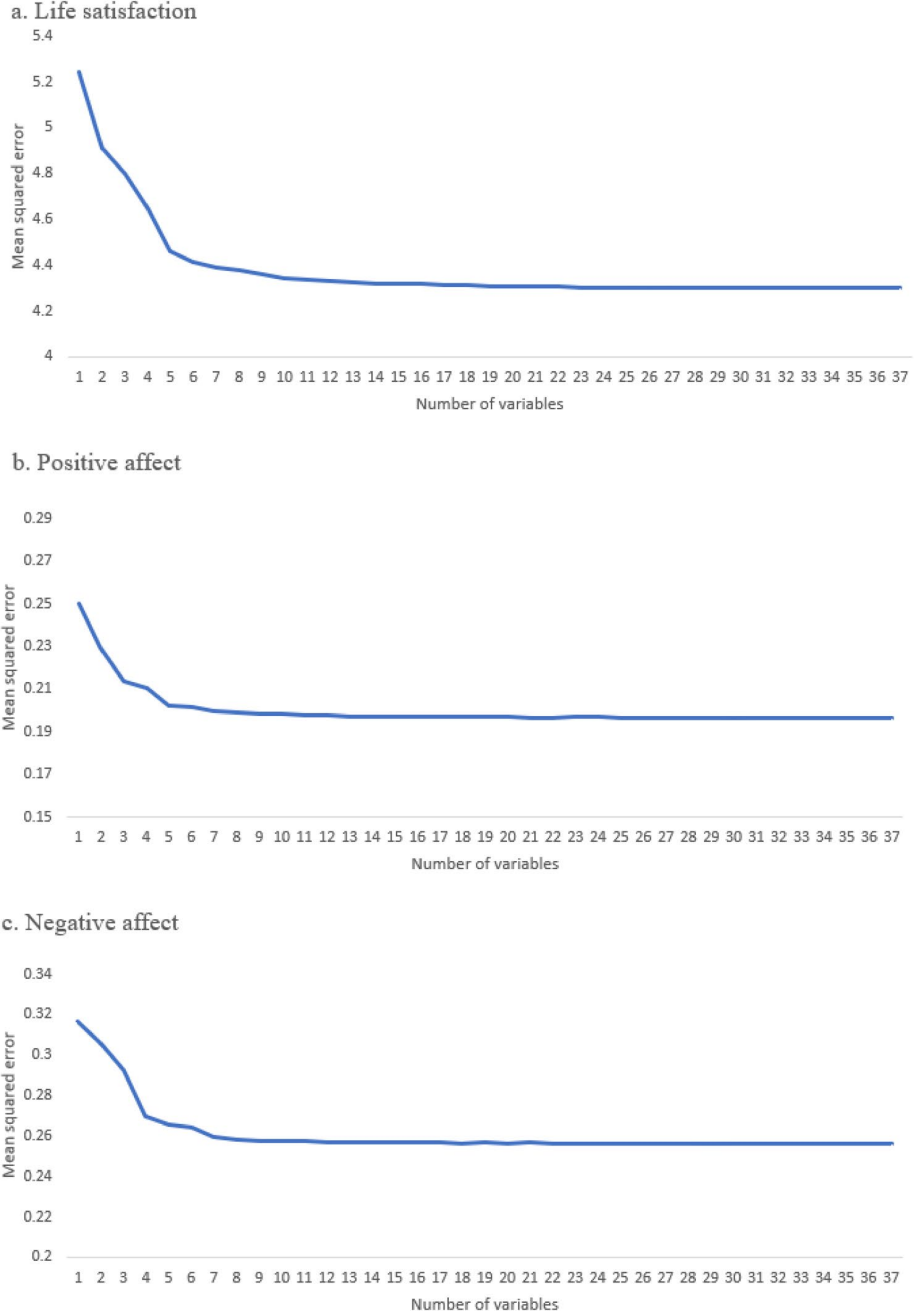
Tenfold cross-validation results.

The LightGBM regression models yield MSE values of 4.248, 0.195, 0.254, and can explain 33.6%, 37.3%, and 29.5% of the variance in life satisfaction, positive affect, and negative affect, respectively. Ten-fold cross-validation was performed. The step-by-step performance of models with an incremental number of factors are shown in Fig. 2. The models with the top 5 predictors had the lowest prediction error (i.e., MSE). This was true for all three dimensions of well-being. The optimal models with the top five factors explained 31.2%, 35.3%, and 26.9% of the variance in life satisfaction, positive affect, and negative affect with MSE values of 4.404, 0.201, 0.263, respectively.

**Figure 3:**
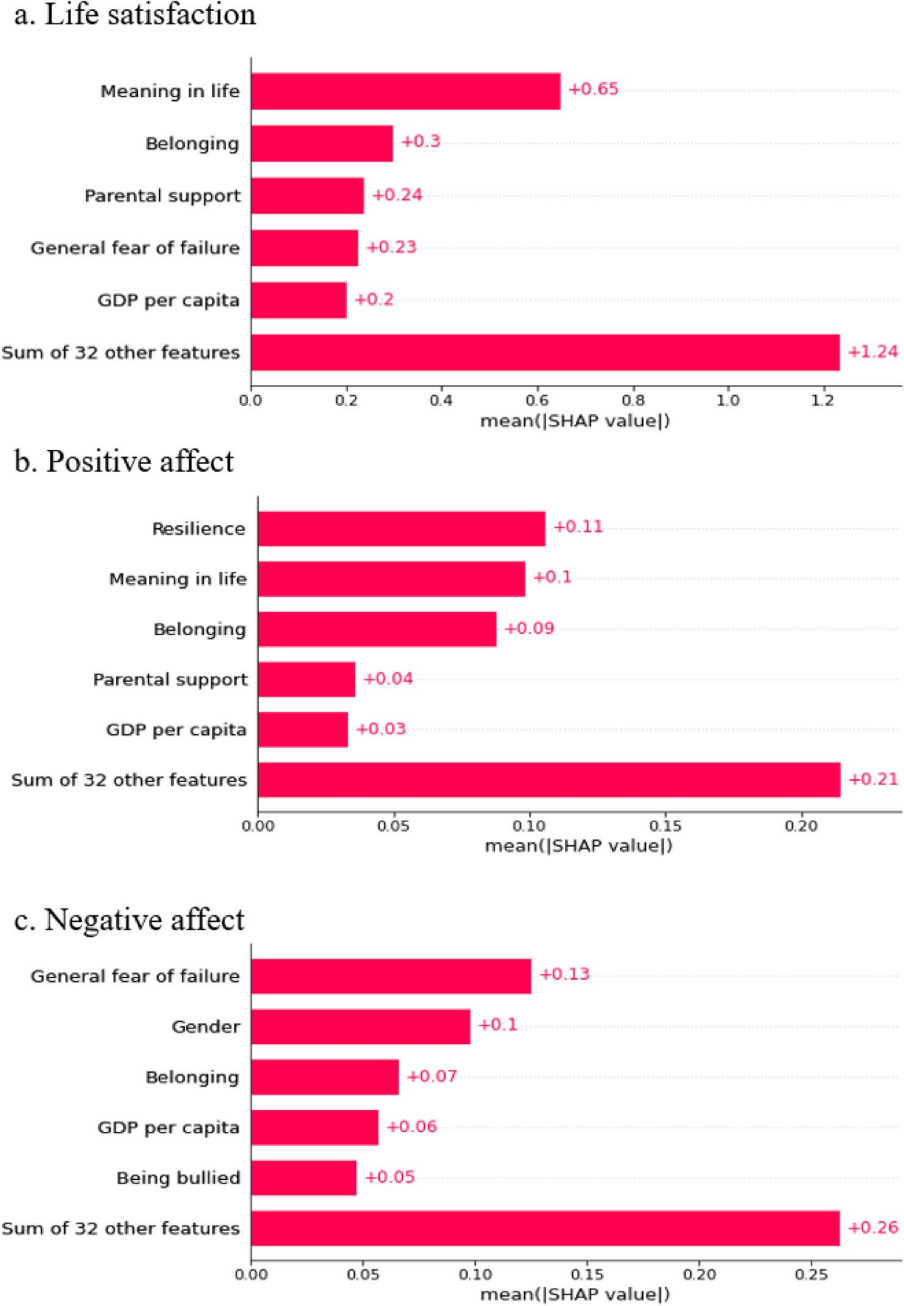
The top predictors of subjective well-being.

Figure 3 shows the ranking of the factors using the SHAP importance plot. Life satisfaction was best predicted by meaning in life, school belonging, parental support, fear of failure, and GDP per capita. Positive affect was most strongly predicted by resilience, meaning in life, belonging, parental support, and GDP per capita. Negative affect was best predicted by fear of failure, gender, experiences of bullying, school belonging, and meaning in life. Table 3 shows top predictors for each culture.

**Table 3:** Top predictors of subjective well-being across cultures.

Cultures	Subjective well-being	Top predictors of well-being							
		Individual			Microsystem			Macrosystem	
Overall sample	Life satisfaction	Sense of meaning	General fear of failure	–	Parental support	Belonging	–	GDP per capita	–
	Positive affect	Sense of meaning	Resilience	–	Parental support	Belonging	–	GDP per capita	–
	Negative affect	General fear of failure	Gender	–	–	Belonging	Being bullied	GDP per capita	–
Western Europe	Life satisfaction	Sense of meaning	Resilience	General fear of failure	Parental support	Belonging	–	–	–
	Positive affect	Sense of meaning	Resilience	General fear of failure	Parental support	Belonging	–	–	–
	Negative affect	General fear of failure	Gender	Resilience	–	Belonging	–	–	GINI
East Central Europe	Life satisfaction	Sense of meaning	Resilience	General fear of failure	Parental support	Belonging	–	–	–
	Positive affect	Sense of meaning	Resilience	General fear of failure	–	Belonging	–	GDP per capita	–
	Negative affect	General fear of failure	Gender	Sense of meaning	–	Belonging	–	GDP per capita	–
East Europe	Life satisfaction	Sense of meaning	Mastery goal	General fear of failure	–	Belonging	–	–	GINI
	Positive affect	Sense of meaning	Resilience	Mastery goal	–	Belonging	–	–	GINI
	Negative affect	General fear of failure	Gender	Sense of meaning	–	Belonging	Being bullied	–	–
Latin America	Life satisfaction	Sense of meaning	Resilience	Gender	Parental support	Belonging	–	–	–
	Positive affect	Sense of meaning	Resilience	Mastery goal	Parental support	Belonging	–	–	–
	Negative affect	General fear of failure	Gender	Sense of meaning	–	Belonging	Being bullied	–	–
English speaking	Life satisfaction	Sense of meaning	Resilience	General fear of failure	Parental support	Belonging	–	–	–
	Positive affect	Sense of meaning	Resilience	–	Parental support	Belonging	Perception of cooperation	–	–
	Negative affect	General fear of failure	Gender	–	–	Belonging	Being bullied	GDP per capita	–
Confucian	Life satisfaction	Sense of meaning	Resilience	General fear of failure	Parental support	Belonging	–	–	–
	Positive affect	Sense of meaning	Resilience	Gender	Parental support	Belonging	–	–	–
	Negative affect	General fear of failure	Gender		–	Belonging	Being bullied	–	GINI
Southeast Asia	Life satisfaction	Sense of meaning	–	–	Parental support	Belonging	–	GDP per capita	GINI
	Positive affect	Sense of meaning	Resilience	Mastery goal	–	Belonging	–	GDP per capita	–
	Negative affect	General fear of failure	Gender	Self-concept: perception of difficulty	–	Belonging	Being bullied	–	–
Africa and the Middle East	Life satisfaction	Sense of meaning	General fear of failure	–	Parental support	Belonging	–	–	GINI
	Positive affect	Sense of meaning	Resilience	–	Parental support	Belonging	–	GDP per capita	-
	Negative affect	General fear of failure	Gender	Sense of meaning	-	Belonging	Being bullied	-	-

Gini pertains to the Gini coefficient which is a measure of income inequality.

### Step 2: conventional statistics

Table 4 shows the parameter estimates and p-values calculated from the HLM analyses. The value of ICC ranged from 0.02 to 0.04 and 0.02 to 0.07 for the school level and the country level, respectively.

**Table 4:** Hierarchical linear models predicting subjective well-being.

Life satisfaction		Positive affect		Negative affect	
Predictors	*β*	Predictors	*β*	Predictors	*β*
(Intercept)	7.32***	(Intercept)	3.23***	(Intercept)	2.65***
Sense of meaning	0.80***	Resilience	0.11***	General fear of failure	0.15***
Belonging	0.38***	Sense of meaning	0.13***	Gender	− 0.23***
Parental support	0.36***	Belonging	0.11***	Belonging	− 0.10***
General fear of failure	− 0.34***	Parental support	0.06***	GDP per capita	0.00^c^
GDP per capita	− 0.00^a^	GDP per capita	− 0.00^b^	Being bullied	0.08***
Random effects					
Within-student residual variance (σ^2^)	4.670		0.214		0.267
Between-country variance (τ00, country)	0.108		0.010		0.021
Between-school variance (τ00, school)	0.176		0.004		0.007
Variance attributable to between-country variation (ICC country)	0.020		0.040		0.070
Variance attributable to between-school variation (ICC _school_)	0.040		0.020		0.020

^a^− 0.0000067, ^b^− 0.00000083, ^c^0.0000014; Gender: Female = 1, Male = 2; ***p < 0.001.

### Supplementary analyses

To explore whether the results across cultures were similar or different, we repeated the LightGBM regression analysis for each of the eight cultural groups. In general, the results in each of the eight cultural groups were broadly consistent with the overall results. More detailed results can be found in the Supplementary Materials (see Table S2).

## Discussion

In this study, we aimed to identify the most important factors predicting students’ subjective well-being globally and across different cultural groups. Rooted in the bioecological theory, our model identified the top predictors of life satisfaction, positive affect, and negative affect. Life satisfaction was best predicted by meaning in life, school belonging, parental support, fear of failure, and GDP per capita. Positive affect was most strongly predicted by resilience, meaning in life, belonging, parental support, and GDP per capita. Negative affect was most strongly predicted by fear of failure, gender, experiences of being bullied, belonging, and meaning in life. Among the different predictors, school belonging and sense of meaning emerged as the most consistent predictor of the different dimensions of subjective well-being.

### Person factors

Consistent with previous studies, this study revealed that girls have higher levels of negative affect^31^. This corresponds to prior research showing that girls are more prone to experiencing negative emotions. However, gender was not a significant predictor of life satisfaction and positive affect.

For psychological factors, sense of meaning, fear of failure, and resilience emerged as key factors. The finding echoed previous studies that showed meaning in life had a positive association with subjective well-being^65^. Meaning in life was often related to the pursuit of life goals, which was positively associated with optimal psychological functioning^66^. Moreover, meaning in life can protect students from the impact of stressful life events^67^.

A positive association between fear of failure and negative affect was found. Fear of failure is a type of avoidance motivation and is closely related to negative feelings, such as guilt, unworthiness, and shame^68^. On the other hand, resilience, defined as the capacity to bounce back in the face of adversity, was found to be positively associated with positive affect, which was consistent with past studies^36,38^. Resilience might be especially important during the adolescent years when students encounter different social problems as they navigate puberty and school transitions.

### Contextual factors

Regarding context, parental support emerged as a crucial factor in predicting subjective well-being. Support from parents can facilitate students’ positive self-evaluations and help them adjust to the school environment effectively^69^.

In terms of the school factors, our results suggest that the sense of belonging in school and experiences of being bullied were particularly important for subjective well-being. These findings also corroborate prior studies^43^. The need to belong is a basic human need^42,70^. Students who feel respected and safe in school tend to engage in school activities with more positive emotions, school satisfaction, and experience less negative emotions^42,71^.

Regarding the experiences of being bullied, our study found a positive association between bullying and negative affect. This finding is consistent with previous studies, which suggested that bullying is a critical negative experience that undermines students’ well-being^17,72,73^. This is an area of concern as bullying might be especially acute in secondary schools^40,74^.

### Implications

This study has several important theoretical and methodological implications. In terms of theory, the current study harnessed the power of a large-scale dataset that involved students from across 71 regions across eight cultural contexts. It provides a comprehensive understanding of the myriad predictors of students’ subjective well-being across the globe. It also extends prior research which has mostly drawn on data from Western cultures. Furthermore, it helps highlight which among the diverse range of factors are most pertinent to predicting and explaining students’ well-being. Although prior studies might have identified certain factors associated with students’ well-being, the novelty of our study was the integrative approach we used. We compared a relatively large number of variables and identified the most powerful and salient predictors.

Methodologically, this study demonstrated the potential utility of combining both machine learning and conventional statistics in data analyses. Our findings suggested different key factors as most important for predicting different dimensions of subjective well-being, indicating the need to simultaneously consider different elements of well-being. Furthermore, it is important to note that not all predictors of well-being are created equal, some have better predictive power than others. However, comparing different well-being predictors in a single study is still relatively uncommon, as most researchers typically focus on the variable they are interested in, neglecting other variables that are also theoretically related to the outcome.

This study also has practical implications and pinpoints several variables that could become intervention targets. We focus on implications for school belonging and meaning, which emerged as consistent predictors of the different well-being dimensions.

Evidence-based interventions can be implemented to promote students’ school belonging. For example, programs that reduce bullying in schools and those that foster cooperative learning and peer tutoring seem to be effective at enhancing school belonging^75^. Furthermore, when teachers show care for their students and create inclusive climates for their classes, school belonging is also enhanced^19^. Specific practices to support belonging could include providing opportunities for student participation, offering constructive feedback, and building positive teacher–student relationships.

Sense of meaning also emerged as a top predictor. Students who see themselves as part of something larger than themselves have a better sense of meaning. Meaning can be fostered through doing volunteer work, being part of extra-curricular activities, and participating in community service. A sense of meaning can also be enhanced when teachers try to help students see the relevance of what they are learning to their personal lives^76,77^. For instance, teachers can state how curricular content can be applied to daily life. They might also encourage students to make explicit linkages between what they are learning in class to their daily lives.

### Limitations and directions for future research

Despite its strengths, this study also has some key limitations. The first limitation is the cross-sectional nature of the PISA dataset. Hence, we are unable to explore the temporal and causal relationships among the variables. For example, is it the case that a higher level of school belonging at Time 1 leads to higher well-being at Time 2 or is the reverse also true? Longitudinal and experimental studies are needed to resolve these questions of directionality and causality.

Second, we only focused on subjective well-being in this study. However, there are dimensions of well-being such as financial, social, and physical well-being^78^. Future studies can also include these other dimensions of well-being.

Third, this study focused on identifying the key predictors of subjective well-being but did not shed light on how these factors relate to or interact with each other. Future studies that explore mediation and moderation mechanisms might be needed to understand the nature of the relationships among the variables.

Last, it should be noted that PISA focuses on adolescent students. Therefore, the findings might only be limited to this developmental stage. Studies that cover other age groups are needed for a fuller account of well-being across developmental stages.

## Conclusion

The present study examined the most important factors that predicted students’ well-being. Across the 37 variables, school belonging and sense of meaning emerged as the most consistent predictors for all three dimensions of subjective well-being. The findings are generalizable across cultural contexts. Perhaps policymakers and educators can take cues from this study to identify potential intervention targets in their attempts to enhance students’ well-being.

### Supplementary Information

Supplementary Table S3.

Supplementary Information.

## Data Availability

This study used the database of 2018 Program for International Student Assessment (PISA) survey that is publicly available from the OECD website (https://www.oecd.org/pisa/data/).
