# Supplementary material for: Identifying the top predictors of student well-being across cultures using machine learning and conventional statistics
Source: Sci Rep. 2024 Apr 10;14:8376. doi: 10.1038/s41598-024-55461-3 (PMC11006657; doi:10.1038/s41598-024-55461-3)
Supplement: Supplementary file 2 — Supplementary Information. [file 41598_2024_55461_MOESM2_ESM.docx]

## Supplementary Materials

### Table S1

### *Comparison of Different Tree-Based Ensemble Machine Learning Algorithms*

| Algorithms | *MSE* | *MAE* | *R^2^* |
| --- | --- | --- | --- |
| **Life satisfaction** | | | |
| LGBMRegressor | 4.248 | 1.557 | 0.336 |
| GradientBoostingRegressor | 4.381 | 1.593 | 0.316 |
| AdaBoostRegressor | 5.187 | 1.849 | 0.19 |
| ExtraTreesRegressor | 4.394 | 1.599 | 0.313 |
| RandomForestRegressor | 4.401 | 1.597 | 0.313 |
| **Positive affect** | | | |
| LGBMRegressor | 0.195 | 0.342 | 0.373 |
| GradientBoostingRegressor | 0.201 | 0.349 | 0.352 |
| AdaBoostRegressor | 0.234 | 0.386 | 0.248 |
| ExtraTreesRegressor | 0.201 | 0.348 | 0.354 |
| RandomForestRegressor | 0.200 | 0.347 | 0.355 |
| **Negative affect** | | | |
| LGBMRegressor | 0.254 | 0.392 | 0.295 |
| GradientBoostingRegressor | 0.266 | 0.404 | 0.262 |
| AdaBoostRegressor | 0.297 | 0.433 | 0.176 |
| ExtraTreesRegressor | 0.263 | 0.399 | 0.268 |
| RandomForestRegressor | 0.260 | 0.396 | 0.276 |

*PS:* MSE = Mean Squared Error; MAE = Mean Absolute Error; R^2^ = The coefficient of determination

### Table S2

### *The Prediction Error (MSE) and Coefficient of Determination (R2) of the Optimal Model with Five Top Predictors for Each Cultural Group*

| Cultures | Overall variables | | Top 5 variables | | Percentage |
| --- | --- | --- | --- | --- | --- |
|  | *MSE* | *R^2^* | *MSE* | *R^2^* |  |
| **Life satisfaction** | | | | | |
| Overall Sample | 4.248 | 0.336 | 4.404 | 0.312 | 92.79% |
| Western Europe | 3.185 | 0.386 | 3.290 | 0.366 | 94.76% |
| East Central Europe | 4.200 | 0.341 | 4.369 | 0.314 | 92.20% |
| East Europe | 4.406 | 0.295 | 4.613 | 0.262 | 88.75% |
| Latin America | 4.076 | 0.367 | 4.322 | 0.329 | 89.61% |
| English speaking | 3.669 | 0.438 | 3.786 | 0.420 | 95.89% |
| Confucian | 4.130 | 0.315 | 4.324 | 0.283 | 89.78% |
| Southeast Asia | 4.601 | 0.214 | 4.744 | 0.190 | 88.65% |
| Africa & Middle East | 6.094 | 0.249 | 6.314 | 0.222 | 89.09% |
| **Positive affect** | | | | | |
| Overall Sample | 0.195 | 0.373 | 0.201 | 0.353 | 94.56% |
| Western Europe | 0.163 | 0.381 | 0.171 | 0.350 | 91.93% |
| East Central Europe | 0.198 | 0.403 | 0.204 | 0.385 | 95.49% |
| East Europe | 0.203 | 0.352 | 0.207 | 0.340 | 96.57% |
| Latin America | 0.176 | 0.380 | 0.185 | 0.347 | 91.38% |
| English speaking | 0.182 | 0.385 | 0.185 | 0.375 | 97.37% |
| Confucian | 0.209 | 0.326 | 0.218 | 0.296 | 90.88% |
| Southeast Asia | 0.198 | 0.298 | 0.206 | 0.271 | 91.02% |
| Africa & Middle East | 0.249 | 0.371 | 0.260 | 0.345 | 93.02% |
| **Negative affect** | | | | | |
| Overall Sample | 0.254 | 0.295 | 0.263 | 0.269 | 90.92% |
| Western Europe | 0.209 | 0.300 | 0.217 | 0.274 | 91.45% |
| East Central Europe | 0.248 | 0.302 | 0.253 | 0.285 | 94.54% |
| East Europe | 0.277 | 0.237 | 0.292 | 0.197 | 83.23% |
| Latin America | 0.242 | 0.278 | 0.248 | 0.261 | 93.74% |
| English speaking | 0.234 | 0.343 | 0.242 | 0.322 | 93.94% |
| Confucian | 0.246 | 0.315 | 0.249 | 0.308 | 97.87% |
| Southeast Asia | 0.255 | 0.166 | 0.266 | 0.131 | 78.74% |
| Africa & Middle East | 0.323 | 0.225 | 0.333 | 0.202 | 90.02% |

## Equation S1

Equations for the HLM Model

***At level 1:***

$$Y_{ijK}=\beta_{0jk}+ \beta_{1jk}(Top 1 variable)+ \beta_{2jk}(Top 2 variable)+ \beta_{3jk}(Top 3 variable)+ \beta_{4jk}(Top 3 variable)+\ldots+r_{ijk}$$

***At level 2:***

$$\beta_{0jk}=\gamma_{00k}+u_{0jk}$$

$$\beta_{1jk}=\gamma_{10k}$$

$$\beta_{2jk}=\gamma_{20k}$$

$$\beta_{3jk}=\gamma_{30k}$$

$$\beta_{4jk}=\gamma_{40k}$$

*…*

***At level 3:***

$$\gamma_{00k}=\pi_{000}+\pi_{001}(GDP per capita)+\pi_{002}(Gini)+e_{00k}$$

$$\gamma_{10k}=\pi_{100}$$

$$\gamma_{20k}=\pi_{200}$$

$$\gamma_{30k}=\pi_{300}$$

$$\gamma_{40k}=\pi_{400}$$

*…*

In the equation at level 1, *Y_ijk_* represents *i*^th^ student’s well-being in *j*^th^ school in *k*^th^ country, *β*_0jk_ was the regression intercept of *j*^th^ school in *k*^th^ country, the values of *β_1jk_* – *β_njk_* represent the fixed effects of the top variables in *j*^th^ school in *k*^th^ country, *r_ijk_* represents the random effect of the *i*^th^ student’s well-being in *j*^th^ school in *k*^th^ country. At level 2, $\gamma_{00k}$ represents the overall well-being of all schools in *k*^th^ country, $u_{0jk}$ was the random effect of overall students in *j*^th^ school in *k*^th^ country. At level 3, $\pi_{000}$ indicates the overall students’ well-being for all schools and all countries, $\pi_{001}$-$\pi_{002}$ represent the fixed effect of country-level factors in *k*^th^ country, $e_{00k}$ refers to the random effect of *k*^th^ country.

## Python Syntax for Data Analysis

### *#Step 1. Machine learning analysis*

**#import packages**

import sklearn.neighbors._base

import sys

sys.modules['sklearn.neighbors.base'] = sklearn.neighbors._base

import pandas as pd

import numpy as np

from sklearn.ensemble import RandomForestRegressor, AdaBoostRegressor, GradientBoostingRegressor,ExtraTreesRegressor

import xgboost as xgb

import lightgbm as lgb

from sklearn.model_selection import train_test_split

from sklearn.metrics import mean_squared_error, r2_score,mean_absolute_error

from sklearn.model_selection import cross_val_score

from missingpy import MissForest

import shap

**# data preprocessing**

# define variables’ name

IVs1 = ['CNTRYID','CNTSCHID','CNTSTUID','ST001D01T','ST016Q01NA','ST186Q01HA','ST186Q03HA', 'ST186Q05HA', 'ST186Q07HA','ST186Q09HA','ST186Q02HA', 'ST186Q06HA', 'ST186Q08HA', 'ST186Q10HA', 'ST004D01T','GRADE', 'AGE', 'BSMJ', 'TMINS', 'STUBMI', 'UNDREM', 'METASUM', 'METASPAM', 'JOYREAD', 'SCREADCOMP', 'SCREADDIFF', 'COMPETE', 'WORKMAST', 'GFOFAIL', 'RESILIENCE','MASTGOAL','EUDMO','EMOSUPS','DURECEC','REPEAT','ESCS','CURSUPP','PRESUPP','SOCONPA','PERCOMP','PERCOOP','ATTLNACT','DISCRIM','BELONG','BEINGBULLIED','DISCLIMA','TEACHSUP','DIRINS','PERFEED','STIMREAD','ADAPTIVITY','TEACHINT','PASCHPOL'] # student-level variables

IVs2 = ['CNTRYID','CNT','CNTSCHID','RATCMP1','RATCMP2','EDUSHORT'] #school-level variables

country = ['GDP_per_capital','GINI'] #country-level variables

#load dataset

stu = pd.read_csv('E:\\EIE-Storage\\E100 Database\\00 PISA\\2018\\CY07_MSU_STU_QQQ.csv', usecols = IVs1) #student dataset

sch = pd.read_csv('E:\\EIE-Storage\\E100 Database\\00 PISA\\2018\\CY07_MSU_SCH_QQQ.csv', usecols = IVs2) #school dataset

cou = pd.read_csv('E:\\EIB-Research\\2022 SHAP SWB_8 cultures\\Country_level.csv') # country dataset

#country code

total = [246, 250, 380, 578, 620, 752, 40, 276, 300, 352, 528, 724, 756, 56, 208, 442, 470,

348, 191, 203, 233, 383, 428, 616, 642, 703, 705, 440, 643,

268,398, 8,70,498, 100, 112, 499,804, 982, 983, 688, 31, 807,

152, 32, 76, 170, 188, 214, 591, 604, 858,

36, 372, 554, 840, 124, 826,

158, 344, 392, 410, 446, 975,

458, 702, 96, 360, 608, 704, 764,

376, 400, 504, 634, 682, 792, 784]

WesternEurope= [246, 250, 380, 578, 620, 752, 40, 276, 300, 352, 528, 724, 756, 56, 208, 442, 470]

EastCentralEurope = [348, 191, 203, 233, 383, 428, 616, 642, 703, 705, 440, 643]

EastEurope = [268, 398, 8, 70, 498, 100, 112, 499, 804, 982, 983, 688, 31, 807]

LatinAmerica = [152, 32, 76, 170, 188, 214, 591, 604, 858]

Englishspeaking = [36, 372, 554, 840, 124, 826]

Confucian= [158, 344, 392, 410, 446, 975]

SouthEastAsia = [458, 702, 96, 360, 608, 704, 764]

AfricaMiddleEast = [376, 400, 504, 634, 682, 792, 784]

#mixed student, school, and country dataset

stu = stu.set_index(['CNTSCHID'])

sch = sch.set_index(['CNTSCHID'])

stu_sch = pd.merge(stu, sch, how = 'inner',left_index = True, right_index = True)

stu_sch.drop(columns = ['CNTRYID_y'], inplace = True)

stu_sch.rename(columns = {'CNTRYID_x':"CNTRYID",'CNT_x':"CNT",'ST016Q01NA':'LS','ST001D01T':'Grade_789','ST004D01T':'Gender'}, inplace = True)

stu_sch = stu_sch.reset_index().set_index(['CNTRYID'])

cou = cou.set_index(['CNTRYID'])

mixed = pd.merge(stu_sch, cou, how = 'inner',left_index = True, right_index = True)

mixed.drop(['CNT','Country','Unnamed: 6', 'Unnamed: 7'], axis = 1, inplace=True)

#replace missing value

missing_value = [' ', 95,96,97,98,99,995,997,998,999,9995,9997,9998.00,9999.00,9999995,9999997,9999998,9999999,99999995,99999997,99999998,99999999

,'95','96','97','98','99','995','997','998','999','9995','9997','9998.00','9999.00','9999995','9999997','9999998','9999999','99999995','99999997','99999998','99999999']

mixed.replace(missing_value, np.nan, inplace=True)

missing_value2= ['5','7','8','9',5,7,8,9]

mixed[['Gender','ST186Q01HA', 'ST186Q03HA', 'ST186Q05HA', 'ST186Q07HA','ST186Q09HA','ST186Q02HA', 'ST186Q06HA', 'ST186Q08HA', 'ST186Q10HA']].replace(missing_value2, np.nan, inplace=True)

#mixed.to_csv('1-mixed_beforeimputation.csv')

#check missing value of each culture

missing_empty = pd.DataFrame(columns = mixed.columns)

for i in range (len(total)):

country = mixed[mixed.index == total[i]]

NA = country.isna().sum()

Total = len (country['CNTSCHID'])

per = pd.DataFrame(NA/Total)

per.rename (columns = {0:total[i]},inplace = True)

per1 = per.transpose()

missing_empty = missing_empty.append (per1)

missing_empty.to_csv('empty.csv')

#check missing value of each country

missing_empty2 = pd.DataFrame (columns = mixed.columns)

cultures = [WesternEurope, EastCentralEurope, EastEurope, LatinAmerica, Englishspeaking, Confucian, SouthEastAsia,AfricaMiddleEast]

for i in cultures:

culture = mixed[mixed.index.isin(i)]

NA = culture.isna().sum()

Total = len (culture['CNTSCHID'])

per = pd.DataFrame(NA/Total)

per.rename (columns = {0:i},inplace = True)

per1 = per.transpose()

missing_empty2 = missing_empty2.append (per1)

missing_empty2.to_csv('empty_culture.csv')

#delete county with high missing value

mixed.drop(columns = ['STUBMI', 'CURSUPP', 'PASCHPOL', 'PRESUPP', 'SOCONPA','TMINS', 'DISCRIM'],inplace=True) #high missing

mixed.drop([807,578,56,208,36,554,124,702,376], inplace=True)#578,56,208,36,554,124,702,376缺少LS; 807 50%的变量都缺失

**#**impute missing value

imputer = MissForest() #we don't use other f

select_country_impute = pd.DataFrame(imputer.fit_transform(culture))

column = culture.columns

select_country_impute.columns = column

**# analysis data with machine learning models**

# split dataset

culture = whole_country

y = culture ['LS'] #DV

X = culture.loc [:,'Gender':'GINI'] # IVs

X_train, X_test, y_train, y_test = train_test_split(X, y, random_state=0)# default_test size = 0.25, random_state

# compare six models

models = [GradientBoostingRegressor(random_state = 0), AdaBoostRegressor (random_state = 0), ExtraTreesRegressor(random_state = 0),RandomForestRegressor(random_state = 0),xgb.XGBRegressor(),lgb.LGBMRegressor()] #define models

for i in range(len(models)):

model = models[i] # select algorithm

model.fit(X_train,y_train) # fit model with training dataset

y_pred = model.predict(X_test) #test model with testing dataset

# Model evaluation

MSE = mean_squared_error(y_test, y_pred)

R2 = r2_score(y_test, y_pred)

MAE = mean_absolute_error(y_test, y_pred)

fp = open('test.txt','a',encoding='utf-8')

fp.write(str(i))

fp.write(str(models[i]))

fp.write(str('MSE='))

fp.write(str(MSE))

fp.write(str('R2='))

fp.write(str(R2))

fp.write(str('MAE='))

fp.write(str(MAE))

fp.write('\n')

# analysis data with the high-performance model

culture = whole_country

y = culture ['PA']

X = culture.loc [:,'Gender':'GINI'] # IVs

X.rename(columns = {"BELONG": "Belonging",

"EUDMO":"Meaning in life",

"EMOSUPS":"Parental support",

"GFOFAIL":"General fear of failure",

"GDP_per_capita":"GDP per capita",

"BEINGBULLIED":"Being bullied",

"RESILIENCE":"Resilience"

}, inplace = True)

X_train, X_test, y_train, y_test = train_test_split(X, y, random_state=0) # split dataset

model = lgb.LGBMRegressor() # define model

model.fit(X_train,y_train) # fit model with training dataset

**#rank variables according to Shapley Additive exPlanations (SHAP) value**

explainer = shap.Explainer (model)

shap_values = explainer (X_train)

Shap_importance = pd.DataFrame(zip(shap_values.feature_names,np.abs(shap_values.values).mean(0)),columns=['feature','importance']).sort_values (by = 'importance', ascending = False)

# Shap_importance.to_csv('AfricaMiddleEast.csv')

order_features = [i for i in Shap_importance['feature']]

num_to_plot = len(order_features)

**# decide the number of top variables using 10-fold cross-validation**

k_scores = []

std_scores = []

i = 0

d = 1

while i < num_to_plot:

index_name = list(order_features[0:i+d])

Xi_train = X_train.loc[:,index_name]

yi_train = y_train

Xi_test = X_test.loc[:,index_name]

yi_test = y_test

model.fit(Xi_train,yi_train)

predicted = np.mean(cross_val_score(use_model, Xi_train , yi_train, cv=10, scoring='neg_mean_squared_error'))

k_scores.append(predicted)

i+=d

mean = pd.DataFrame (k_scores, columns = ['Mean'])

# mean.to_csv('AfricaMiddleEast_com.csv')

**# bar plot of the top five variables**

topi = 5

shap_values.base_values = shap_values.base_values[:,].flatten()

select_feature = order_features[0:topi]

rank_no = Shap_importance[Shap_importance['feature'].isin(select_feature)].index

# Plot shap summary plot

shap.plots.bar(shap_values,max_display=6)

### *#Step 2. Hierarchical linear modelling (HLM) analysis*

# import library

library(lme4)

library (jtools)

library(lmerTest)

# 2-level HLM model

m1 <- lmer (PA ~ SCHLTYPE+STRATIO+TEACHINT+QUALIFICATION+ESCS+CLSIZE+STUBEHA+DIRINS+STIMREAD+(1|CNTSCHID), data = data, REML = FALSE)

summary (m1, digits=10)

summ(m1, digits=10)

# 3-level HLM model

m2 <- lmer(PA~ EUDMO+BELONG+EMOSUPS+GFOFAIL+GDP_per_capita+(1|CNTSCHID)+(1|CNTRYID), data = data, REML = FALSE)

summary (m2, digits=10)

summ(m2, digits=10)
